# The Tendon Jerks

**Published:** 1904-07-09

**Authors:** 


### Hospital Clinics.

### THE TENDON JERKS.

The recognition and clinical interpretation of the so-called tendon phenomena are among the events of comparatively modern medicine, but they have been sufficiently long the subjects of professional study to secure that the medical practitioners of the present generation accept them as an essential part of a routine examination of the individual patient. Speaking generally, it may indeed be said that the physiology of the tendon jerks is not in some respects in as secure a position as might be desired. But this hardly attaches any qualification to the practical significance of those departures from the normal that are to be observed in the study of disease. To present the physiological doctrine in broad terms, it may be accepted as a fairly well-established conclusion that, though the tendon jerks are not true reflexes, their occurrence depends on the integrity of certain reflex arcs. The movement of the leg, for example, when a blow is delivered on the patella tendon is not in the proper sense of the term a reflex movement—that is, it is not due to a discharge from a nerve centre excited by a nervous impression originated by the blow and reaching the spinal cord along an afferent nerve. On the contrary, it is the result of a contraction of the muscle directly excited by the stimulation of its fibres by the sudden increase of tension produced by the blow on the tendon. The knee-jerk may thus be presented as essentially a direct contraction of the muscle following a special form of local stimulation. At the same time, it must be recognised that the ability of the muscle to respond to this variety of stimulus is dependent upon a certain state or condition of the muscular tissue, and this state or condition, in turn, only exists when the muscle retains its organic connection with the nerve-centres through the efferent fibres of the spinal system of nerves. This, however, is not all. Not only must there be the connection just defined, but, in addition, there must exist an unbroken afferent path from the muscle to the spinal centres. Thus there is formed a reflex arc, consisting of afferent fibres from muscle to spinal cord, spinal centre, and efferent fibres from this centre to terminate in the muscle. Any break in this arc removes from the muscle that nervous influence upon which the capacity of the muscle to respond to a sudden increase in the tension of its tendon depends. Hence, though not a reflex movement, the knee-jerk—and the same is true of other tendon jerks — is conditioned by a reflex apparatus; and a flaw in that apparatus will produce exactly the same consequences as would follow were the fact of the knee-jerk a true reflex movement following an afferent impression, a central explosion, and an efferent discharge. As a consequence, it is readily seen what various circumstances will abolish the knee-jerk. First, the afferent path of the reflex mechanism may be interrupted, as, for example, by division of the posterior (sensory) root of the corresponding spinal nerves or, as occurs more frequently in practice, by sclerosis of the afferent nerve-fibres after they have entered the cord—that is, in the posterior columns (tabes dorsalis, hereditary ataxia, general paralysis). The same result will follow destruction of the corresponding nerve-centres situated in the anterior cornu of the cord and thus may appear in a transverse myelitis or in infantile paralysis (acute anterior polio-myelitis). Again, the efferent tract in the reflex pathway may be broken, as when peripheral neuritis affects the lower limbs. Therefore, absence of the knee-jerks may be noted in diphtheritic paralysis, in alcoholic neuritis, in diabetes; and it is worthy of note that the several poisons on which these diseases depend find the reflex mechanism of the knee-jerk a highly susceptible point in the nervous apparatus. This is shown by the fact that in these conditions there may be loss of the knee-jerks with little or no other evidence of prejudicial action on the peripheral nerves. Finally, to complete the list of circumstances which weaken or destroy the knee-jerk, must be mentioned disease of the quadriceps extensor muscle itself. It is, of course, obvious that if the muscular fibres are atrophied, or in a condition of advanced mal-nutrition, there can be no muscular response to a local stimulus, and hence the knee-jerk is lost when pseudo-hypertrophic paralysis or other form of myopathy affects the substance of the quadriceps extensor. All this is perfectly plain sailing. To put it in a word, it comes to this: that if the reflex arc conditioning the knee-jerk is broken, or the quadriceps muscle is seriously diseased, the knee-jerk is diminished or lost. The doctrinal explanation is less satisfactory when the conditions which lead to exaggeration of the knee-jerk are considered. The general statement is that the cerebral centres exercise through some of the fibres of the upper motor neurons (pyramidal tracts) an inhibitory or restraining influence upon the spinal centres associated with the tendon jerks, and that removal of this influence leads to exaggeration of these jerks. Certain it is that many lesions which interrupt one or both of the pyramidal tracts are indicated by exaggeration of the tendon phenomena. This result is seen, therefore, in sclerosis of the lateral columns—spastic paraplegia, disseminated sclerosis, amyotrophic lateral sclerosis, syringomyelia. The same fact is to be observed, on one side of the body, in hemiplegia, and it would seem that a lesion which produces little or no other evidence of an interruption in the continuity of the pyramidal tract may be quite sufficient to cause an appreciable exaggeration of the tendon jerks. This is illustrated in cases of hemiplegia in which after recovery from the paralysis a heightened knee-jerk and an extensor response of the big toe (Babinski's sign) still remain. The same facts in other cases may be the only grounds on which to rest a diagnosis of an intracranial lesion. A middle-aged man has perhaps a slight attack of giddiness, or other similar sensation, and for a few hours is said not to have walked very well. Examination may detect nothing save the facts just mentioned, yet they are ample, and their recognition indicates without uncertainty the necessary diagnosis. Whatever the theoretical explanation, there can be no question of the definiteness and precision with which an exaggeration of the tendon jerks points to a lesion in the upper motor neurons (cerebro-spinal segment). The "lesion" may, it is true, be functional, at least to the extent that in hysteria the knee-jerks are often very conspicuous, and there may even be some measure of ankle-clonus. But if hysteria can be excluded—and Babinski's sign may be held to effect this—the exaggeration means without question an organic change in the central nervous system somewhere above the physiological level of the spinal centres. If this were all, there would be no need to scrutinise too closely the suggested explanation that such exaggeration is explained by removal of the inhibitory influence of the cerebral centres. But it is not all. On the contrary, there is experimental and clinical evidence to show that complete transverse division of the cord, so far from producing exaggeration of the tendon jerks, as would be expected from the above hypothesis, is followed, as a matter of fact, by exactly the opposite result. Again, there are various intracranial conditions which produce not excess but defect of the knee-jerks. This has been noted more particularly in connection with tumours of the cerebellum, but it is not confined to these. It has been associated with growths of the pre-frontal lobe, and also with tubercular meningitis. Even this statement does not exhaust the possible modifying effects of intracranial lesions on the condition of the tendon jerks, as in some cases of cerebellar tumour these have been noted as exaggerated. In some of these latter the explanation is doubtless to be found in the shape of pressure on the pyramidal tracts where these pass through the pons and medulla, but in others this suggestion is not available. One of the most interesting explanations of the effect of intracranial lesions on the tendon phenomena is that advanced by Hughlings Jackson. According to this view the motor cells in the anterior cornua of the spinal cord receive streams of energy both from the cerebral cortex and from the cerebellum. The cerebral influence tends to restrain the activities of these cells, the cerebellar to increase these activities. That is, the cerebral centres give rise to nervous impressions which diminish such phenomena as the knee-jerk, whilst the cerebellar influence is in the direction of exaggerating such phenomena. When the centres of the cord are cut off from those of the cerebral cortex, as by a lesion in the pyramidal tract, they are left to the unantagonised influence of the cerebellum, and the tendon jerks are therefore in excess. On the other hand, when the influence of the cerebellum is destroyed, as by a cerebellar tumour, the inhibitory cerebral tendency is unchecked, and the tendon jerks are consequently diminished or absent. Whether this covers all the facts may perhaps be doubted, though strong arguments may be advanced in its support. In so far as there is uncertainty there is a corresponding need for further clinical and pathological investigation. But the important practical point is obtained by a recognition of the fact that a modification of the tendon jerks may be due to a purely intracranial condition. Unilateral excess may be the sole objective evidence of a lesion in one of the pyramidal tracts, whilst with tumours of the cerebellum or pre-frontal lobe there may be defect or suppression, this being also sometimes seen in basal meningitis; in other cerebellar growths the tendon jerks may be exaggerated. The explanation of these facts is difficult, and perhaps at present impossible, but it may fall to the lot of any careful observer to secure the opportunity which will resolve the mystery.

